# A time motion study of community mental health workers in rural India

**DOI:** 10.1186/s12913-019-4732-7

**Published:** 2019-11-21

**Authors:** Vijaya Chebolu-Subramanian, Nachiket Sule, Richa Sharma, Nerges Mistry

**Affiliations:** 1 IFMR Graduate School of Business, KREA University, 5655, Central Expressway, Sri City, Andhra Pradesh 517646 India; 2 Foundation for Research in Community Health, 3-4, Trimiti-B Apartments, Anand Park, Aundh, Pune, Maharashtra 411007 India

**Keywords:** Time motion study, Community health workers, Mental health program, Work flow, Value stream mapping

## Abstract

**Background:**

Community Health Workers (CHWs) are critical to providing healthcare services in countries such as India which face a severe shortage of skilled healthcare personnel especially in rural areas. The aim of this study is to understand the work flow of CHWs in a rural Community Mental Health Project (CMHP) in India and identify inefficiencies which impede their service delivery. This will aid in formulating a targeted policy approach, improving efficiency and supporting appropriate work allocation as the roles and responsibilities of the CHWs evolve.

**Methods:**

A continuous observation Time Motion study was conducted on Community Health Workers selected through purposive sampling. The CHWs were observed for the duration of an entire working day (9 am- 3 pm) for 5 days each, staggered during a period of 1 month. The 14 different activities performed by the CHWs were identified and the time duration was recorded. Activities were then classified as value added, non-value added but necessary and non-value-added to determine their time allocation.

**Results:**

Home visits occupied the CHWs for the maximum number of hours followed by Documentation, and Traveling. Documentation, Administrative work and Review of work process are the non-value-added but necessary activities which consumed a significant proportion of their time. The CHWs spent approximately 40% of their time on value added, 58.5% of their time on non-value added but necessary and 1.5% of their time on non-value added activities. The CHWs worked for 0.7 h beyond the stipulated time daily.

**Conclusion:**

The CHW’s are “dedicated” mental health workers as opposed to being “generalists” and their activities involve a significant investment of their time due to the specialized nature of the services offered such as counselling, screening and home visits. The CHWs are stretched beyond their standard work hours. Non-value added but necessary activities consumed a significant proportion of their time at the expense of value-added activities. Work flow redesign and implementation of Health Management Information Systems (HMIS) can mitigate inefficiencies.

## Background

Several countries in the developing world face challenges in the delivery of quality health care services due to a human resource deficit [1]. Imbalances in the availability of skilled personnel and inequalities within the distribution of a country’s health work force also contribute to the deficit, with fewer health care providers attending to rural areas as compared to urban areas [1–3].

Countries such as India, Brazil, Nepal and Bangladesh are attempting to bridge this gap and facilitate health care delivery with cadres of workers known as Community Health Workers (CHWs) [4, 5].

In India, one of the key thrust areas of the National Rural Health Mission is to improve the availability of critical workforce in rural areas through the Community Health Worker initiative. The CHWs are generally incentivized volunteers who are imparted limited formal training and work in communities to enable the extension of health systems and services to resource constrained and low access areas [6, 7].

The Community Mental Health Program (CMHP) run by the Foundation for Research in Community Health (FRCH) is one such program which utilizes CHWs to provide health services in rural areas. The primary intent of the CMPH is to respond to the large gap in mental health care through the delivery of a range of appropriate interventions to persons with selected mental disorders.

The project is designed on the lines of a task-shifting model wherein the scarcity of health care personnel is addressed by shifting some of the tasks of a psychiatrist and a psychologist to the primary care doctor and CHWs.

The CMHP has four functional CHWs and as the program evolved it was observed that there was a steady increase in their work load and a gradual scaling up of their duties. The CHW’s were taking on greater roles and responsibilities as they gained more expertise with experience. It was therefore felt that there is a need to undertake a study to understand the work flow and burden of the CHWs and its impact on service delivery.

Time Motion study is a methodology in the which the time duration of every activity of a subject is recorded to establish the work flow and ensure efficiency and effectiveness through the elimination of waste and simplification of work [8–10]. Time Motion studies have been used extensively in healthcare to quantify the time utilization and work flow of pharmacists, physicians and nurses in hospitals and clinics [11–21].

In contrast, studies at the community level are fewer in number. Odendaal and Lewin (2014) state that this may be because CHWs are often seen as not being a part of the formal health system and secondly it is extremely challenging to conduct these studies at the field level [22]. They conduct a study in Cape Town on CHWs providing treatment and adherence support to people on Tb treatment. Jeffries et al. study health workers in Ethiopia working on a health extension program to understand how their time allocation [23]. Philips et al. (2014) analyze community patient navigators in DuPage County and report that they could spend more time on communicating and engaging with the patients if their documentation responsibility is reduced [24]. Singh et al. asses the time utilization factor of health workers from South India [25, 26]. They find the auxiliary midwives to be overworked and suggest a reassessment of the workload.

Limited studies at the community level is especially a major gap in Indian health services research. The services offered by these workers in a country like India are critical and studies enabling formulation of a targeted approach to human resource planning are needed. Therefore, to address this gap, understand the feasibility issues of undertaking such a study at the field level and record the work flow of the CHWs in the CMHP, we undertake a Time motion study.

## Methods

### Setting

The study was conducted between November and December 2015 in the CMHP program which is active across six sites in India including the rural area of Pune district in the state of Maharashtra and has four functional CHWs. These months were chosen as the weather is fairly moderate during this period with minimal extreme variations. The program is implemented in the work area of two Public Health Centers (PHCs) serving a population of 55,000 in 29 villages. Two of the CHWs serve a population of 23,000 while the other two cater to the remaining 32,000. The connectivity through roads and transport (public and private) is average to poor in areas served by the program. Farming is the primary occupation of the people followed by employment in local factories.

Criteria for selecting the CHWs was greater work experience, familiarity with the work agenda, grasp of the processes and structure of work and understanding of the nuances of the implementation Moreover, the CHWs had a similar mode of transport (a two-wheeler vehicle).

### Data collection

Few classifications especially for CHWs activities in the field could be found in literature and those found were not directly applicable [22–26]. The FRCH during the initiation of the CHWS defines their roles and responsibilities as:

1. Identifying patients with priority mental health disorders
2. Counselling and following-up of identified patients
3. Conducting awareness meetings in the community
4. Screening patients
5. Coordinating with family members of identified patients
6. Referral of patients
7. Documentation and reporting of all activities in the prescribed format

We conducted extensive discussions with the FRCH staff, coordinators of the CMHP program and CHWs to understand the current activities of the CHWs and develop a work classification system. Further, a pilot run was conducted prior to the actual study for a period of 3 days to check for feasibility of the study and refine the recording sheets to be used. Based on this, the classification system was continually refined to add more granularity to the categorization and define the activities clearly (Table 1).

**Table 1:** Activities performed on different days, Mean and Median time per activity

	Activity	Mean Time (minutes)	Median Time (minutes)	OPD day	Field day	Review meeting day
1	Administrative work	12.98	6	Yes	Yes	Yes
2	Counselling	19	17	Yes	Yes	
3	Doctor consultation	7.22	3.5	Yes		
4	Patient interaction	6	3.5	Yes		
5	Home visits	31.53	25		Yes	
6	Screening	9.38	8.5	Yes	Yes	
7	Staff interaction	4.75	3.5	Yes	Yes	
8	Community interaction	23.17	12.5		Yes	
9	Review of work process	61.71	55			Yes
10	Travelling	12.11	5	Yes	Yes	Yes
11	Documentation	44.86	56	Yes	Yes	Yes
12	Break	20.87	18	Yes	Yes	Yes
13	Waiting for patient arrival	3	3		Yes	
14	Miscellaneous	11.8	5	Yes	Yes	

The study was a continuous Time- Motion study. The data was collected and recorded by an observer (a researcher) external to the organization to neutralize any personal bias. The observer “shadowed” the CHWs continuously on any given day and recorded the time duration of the activities with a stop watch. The observer manually noted the data on the pre-designed and pre-tested recording sheet (Additional file 1). At the end of the day, the data was computerised and transferred to an excel sheet. For the purpose of triangulation, the daily logs maintained by CHWs were cross checked against the data recorded and the agreement was acceptable.

It was identified that there are three major components in the CHW’s work schedule: Field work, Out Patient Department (OPD) and Review meetings. During Field days, CHWs identified and screened patients by conducting home visits, increased awareness about mental health and counseled patients. On OPD days, the CHW was present at the Public Health Centre during the psychiatrist’s visit for consultations. On review meeting days, monthly activities, complex cases, reporting, weekly work and difficulties encountered in the field were discussed.

On any given day a CHW performed only one of these work components. The CHWs worked for 26 days in a month which comprised of 20 days of Field work, 2 days of OPD and 4 days of Review meetings.

Each CHW was “shadowed” by the observer for the duration of an entire working day (9 am- 3 pm) for 5 days, staggered during a period of 1 month. The 5 days were randomly selected to include three Field work days, one OPD day and one Review meeting day so as to ensure the recording of the entire work flow of the CHWs. It was found that the CHWs performed any or all of 14 independent activities during any given day (Table 1). For detailed activity definitions refer to Additional file 2.

### Ethical considerations

The study was approved by the Institutional Research Ethics Committee (IREC) at FRCH (IREC/2015/11/4/1). The committee approved post facto consent to reduce bias in the study sample. All participants (CHWs) provided verbal consent prior to the start of the study and no identifiers were collected. Post-facto written consent was then obtained from the study participants. To maintain confidentiality of the patients and their families, the observer was made to sign a declaration form.

### Data analysis

One of the main objectives of this study was to analyse whether the CHWs were able to allocate adequate time to their primary role or were over-burdened with activities which were extraneous to the key components of the program. This was particularly important in the view of the total population size that they covered.

To this end, after extensive discussions with the program personnel, we categorized the work activities performed by the CHWs into three categories (Table 2) based on principles of value-stream mapping, a methodology used to continuously improve process quality and efficiency by eliminating waste [11, 27, 28].

I. Value added activities: Patient centred activities which constitute the primary roles and responsibilities of the CHWs and reflect the principal intent of the CMHP.
II. Non-value added but necessary activities: Activities supporting the conducting of the value-added activities.
III. Non-value- added activities: Non-essential to the functioning of the CHWs.

**Table 2:** Activity categorisation

	Value added Activities	Non-value added but necessary Activities	Non-value-added Activities
1	Home visits	Documentation	Miscellaneous
2	Community interaction	Review of work process	Waiting
3	Counselling	Administrative work	
4	Screening	Staff interaction	
5	Doctor consultation	Traveling	
6	Patient interaction	Break	

The type of activity observed and it’s time duration with other details were individually recorded for each CHW and the aggregate time duration and work flow was analysed. The goal was to calculate the % time spent on Non-value added but necessary and Non-value-added activities and compare it with the time spent on Value added activities. The distance travelled by the CHWs for performing an activity (if any) and the time taken for travel, terrain type and the mode of transport utilized were also recorded.

## Results

### Program activities

**Fig. 1:**
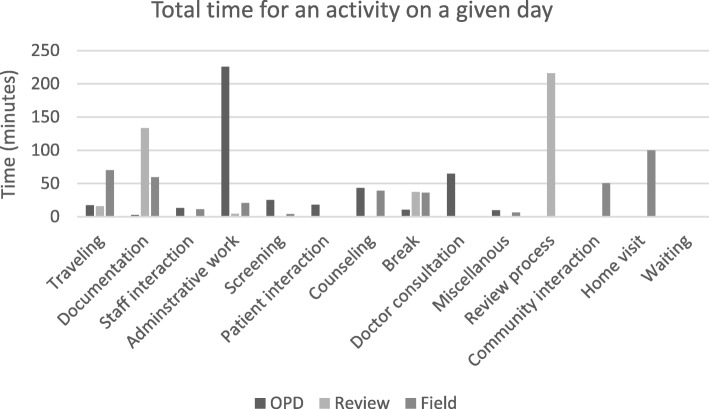
Total time for an activity on a given day

The workload analysis is for the current population size being served by the CHWs. It was found that the CHWs work for an average of 174 h in a month. They perform more activities on the Field and OPD days as compared to the Review meeting days. The mean and median time per activity were calculated (Table 1). On an OPD day the CHWs on an average spent the most amount of time i.e. 226 min on Administrative work followed by 65 min on Doctor consultation. On a review meeting day, Review of work process occupied them for 216 min followed by Documentation for 133.5 min. A field day involved 99.83 min of Home visits followed by 70.33 min of Traveling (Fig. 1).

**Fig. 2:**
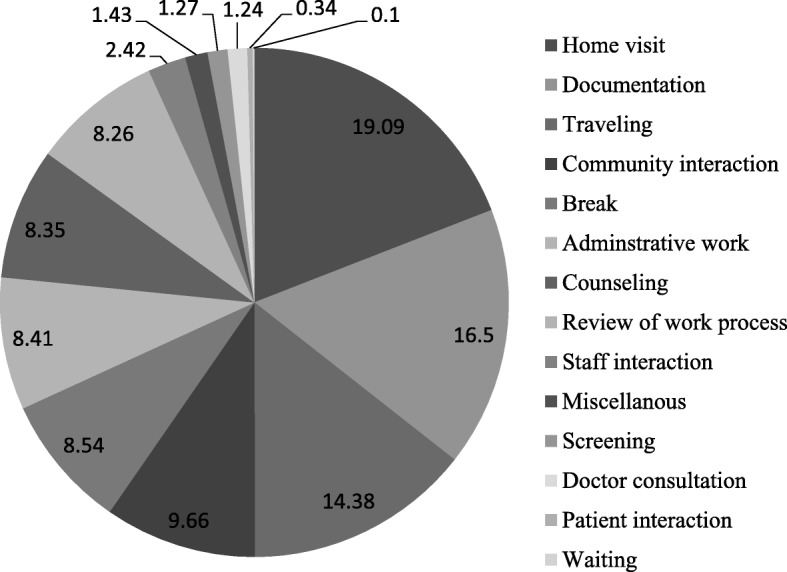
Monthly work breakdown of activities for a CHW (% of total work hours)

The monthly work breakdown of the CHWs shows that Home visits occupy them for the maximum time, followed by Documentation, Traveling and then Community interaction. They spend approximately equal amounts of time on the next three activities of Breaks, Administrative work and Counselling (Fig. 2).

**Fig. 3:**
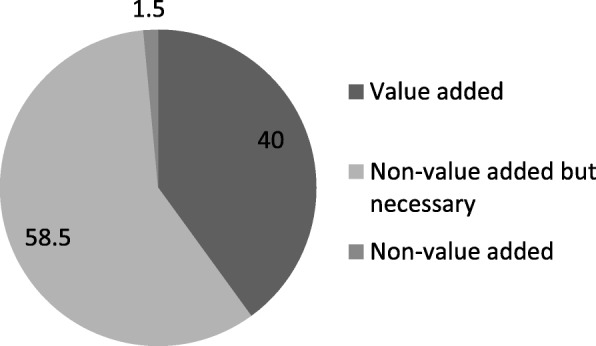
Monthly total work time breakdown (%) according to Value stream mapping

It can be seen (Fig. 3) that the CHWs spend approximately 40% of their total time on value-added activities and 58.5% on non-value added but necessary activities. The non-value-added activities occupy approximately 1.5% of their total time on an average.

### Travel and terrain

In a month a CHW travels approximately 355 km of which 327 Km are on Field days, 10 Km on OPD days and 18 Km on Review meeting days. The CHWs either walk or use their own transport (2-wheeler vehicle). Also, approximately 94% of the travel utilizes the vehicle and only 6% involves walking.

We qualitatively categorized the terrain covered by the CHWs as “Easy”, “Moderate” or “Difficult” based on ease of navigation. It was seen that approximately 68% of the total travel is across “Easy” terrain while 27% is across “Moderate” and 5% across “Difficult” terrain. To study the impact of terrain, we also compared the speed of travel across terrain but no significant difference was observed.

## Discussion

Our study found that the CHWs broadly perform 14 independent activities as part of their work responsibilities. We categorized 6 of these activities as value added activities based on the initial roles and responsibilities assigned to them by FRCH, additionally 6 activities are non-value added but necessary activities and 2 are non-value-added activities. It was observed that Documentation was the only non-value added but necessary activity described in their initial duties list.

Home visits occupy them for the maximum time, followed by Travelling, Documentation and Community interaction. They spend approximately equal amounts of time on the next four activities of Break, Administrative work, Counselling and Review of work process.

The CHWs spent approximately 40% of their time on value-added activities, 58.5% of their time on non-value-added but necessary activities and 1.5% of their time on non-value-added activities. Our discussions with the team revealed that the CHW’s worked approximately 2–3 h beyond the standard work hours in a week and that these extended hours were mainly complete documentation. This is captured in our study with the average daily work duration being 6.7 h with Documentation being a major activity. This indicates that the CHW’s are already stretched beyond their stipulated hours and are unable to devote requisite time to core program activities.

Though Documentation is a required responsibility it consumes a relatively large proportion of their time along with Administrative work and the Review of work process. They spend a significant amount of time on Administrative work on OPD days. These activities can be reviewed to gauge the pressure points that the CHWs experience while performing them and steps can be taken to mitigate them by redesigning the workflow. For example, duplication of effort between departments and data sets or issues in quantifying the outcome of care provided can be analysed. Evolving regulatory and policy requirements for these activities may also exacerbate these problems and need to be assessed.

Traveling on the vehicle especially during Field days takes a major chunk of time, and frequent breakdown of vehicles were also reported, hence provision for better vehicles can mitigate this issue.

Individual differences between CHWs were also observed based on their personality as some thrived during community interactions. Workload also increased when the CHW was assigned to villages closer to the clinic as overall patient enrolment was higher from these villages.

The CHW’s in this study are completely “dedicated” to the mental health program in contrast to other community health workers such as the Accredited Social Health Activist (ASHA) who are “generalists” and offer more routine, standardized services in the areas of maternal and child health. Hence, the CHW activities in CMHP such as counselling, screening and home visits involve a significant investment of their time due to the specialized nature of the services offered. Therefore, segregation of the “generalist” and “dedicated” health worker is necessary and a single tier “generalist” cadre of workers may not be able to address the requirements arising due to varied healthcare challenges.

The design and implementation of a Health Management Information System (HMIS) to record retrieve and process data can also greatly contribute towards reducing inefficiencies in the system. For program managers, a HMIS also provides a better tool for monitoring and supervision and data management [29].

### Limitations

One of the major challenges and possible limitations of the results is the effect of “shadowing” on the CHWs. To lessen this effect, the observer was taken to the field and other program areas a few times to get the CHWs acclimatized to his presence. He was also taken to another program area to alleviate any suspicion. Surrogates in the form of a Programme Coordinator, Research Associates and a Research Officer also visited the field to divert attention from the observer.

Since the CHWs are involved in numerous activities, a month’s duration is not sufficient for a distance and terrain analysis. A longer time period (3–4 months) is required to conduct any such analysis. The extended period will also aid in getting the CHWs acclimatized to external presence.

The observer reported that the CHWs work is affected to a significant extent by weather conditions such as extreme heat. A very hot day makes them spend less time on the field and more time on documentation or at the office whereas a pleasant day extends the hours they spend on field. A longer duration study will enable tracking the impact of weather conditions.

## Conclusion

Despite the limitations of the current study we hope this work will be a precursor for more extensive projects with larger sample sizes to study the work flow of Community Health Workers present at the grassroots level and inform on the quantum of work load that they can undertake. This we believe, will aid policy makers in human resource planning, development of training episodes, devising of adequate support systems and formulation of compensation mechanisms for the continued successful implementation of these programs.

## Supplementary information

**Additional file 1:** The data recording sheet utilized for the Time Motion study.

**Additional file 2:** Definitions of the work activities performed by the CHWs.

## Data Availability

The datasets used and/or analyzed during the current study are available from the corresponding author on reasonable request.

## Abbreviations

CHW: Community Health Worker
CMHP: Community Mental Health Program
FRCH: Foundation for Research in Community Health
HMIS: Health Management Information Systems
OPD: Out Patient Department
PHC: Public Health Centre
