# Supplementary material for: A time motion study of community mental health workers in rural India
Source: BMC Health Serv Res. 2019 Nov 21;19:878. doi: 10.1186/s12913-019-4732-7 (PMC6873675; doi:10.1186/s12913-019-4732-7)
Supplement: Supplementary file 1 — Additional file 1: The data recording sheet utilized for the Time Motion study. [file 12913_2019_4732_MOESM1_ESM.docx]

**Data Recording Sheet**

The sheet included provisions for recording the following data:

1. Serial Number
2. Name of the CHW
3. Date
4. Work component (Field, Review, OPD)
5. Type of activity and sub-activity
6. Start and End Time of each activity
7. Location: Start and End location (Village, Hamlet name)
8. Mode of Transportation (if any)
9. Distance travelled (if any)
10. Terrain
11. Frequency of transportation (if applicable)
12. Remarks: of the observer (if any)
