# Supplementary material for: A time motion study of community mental health workers in rural India
Source: BMC Health Serv Res. 2019 Nov 21;19:878. doi: 10.1186/s12913-019-4732-7 (PMC6873675; doi:10.1186/s12913-019-4732-7)
Supplement: Supplementary file 2 — Additional file 2: Definitions of the work activities performed by the CHWs. [file 12913_2019_4732_MOESM2_ESM.docx]

**CHW’s Work Activity Definition**

1. **Administrative work**: This activity includes all administrative tasks such as organizing the OPD, managing files and stationery, regulating patients in the OPD, patient registration at OPD, logistics arrangement (furniture, room) etc.
2. **Counselling:** This activity involves the psychosocial support component of the programme and these sessions are conducted with the patients and their family members.
3. **Doctor consultation**: The CHWs accompany the patient to the doctor’s room to discuss the case history and treatment plan.
4. **Patient interaction**: During this activity CHWs meet the patients and talk as a form of rapport building.
5. **Home visits**: Home visits are conducted by the CHW in the field and include visiting the home of the patient or community member with the purpose of spreading awareness about mental health disorders and the programme and identifying probable patients
6. **Screening**: During this activity the CHWs use a tool to screen patients for probable symptoms of Common Mental Disorders and assess their status according to the results.
7. **Staff interactions**: Interactions that take place between the coordinators and the CHWs or among the CHWs themselves regarding patients, care takers or any aspect of the programme are classified under this activity.
8. **Community interaction**: This activity includes awareness meetings conducted by the CHWs for groups of people in the community.
9. **Review of work process**: The review meetings are held once every week and are attended by the coordinators and all the CHWs. Complex cases, reporting, weekly work and difficulties encountered in the field are discussed in these meetings.
10. **Travelling**: Travel by the CHWs to reach office or the PHC as well as travel during field work day or under any other circumstance.
11. **Documentation:** CHWs’ document their activities in a daily log in the prescribed format.
12. **Break**: Lunch, tea, washroom breaks etc.
13. **Waiting:** This is usually the time during which the CHWs wait for people to assemble for a community meeting or for the patient/care taker to be available for a session.
14. **Miscellaneous:** Miscellaneous activities include everything occurring outside the ambit of the programme but during the working hours such as tending to a vehicle needing repair, visit to the petrol station etc.
